# A Systematic Literature Review for Blockchain-Based Healthcare Implementations

**DOI:** 10.3390/healthcare13091087

**Published:** 2025-05-07

**Authors:** Mutiullah Shaikh, Shafique Ahmed Memon, Ali Ebrahimi, Uffe Kock Wiil

**Affiliations:** SDU Health Informatics and Technology, The Maersk Mc-Kinney Moller Institute, University of Southern Denmark, 5230 Odense, Denmark; shaf@mmmi.sdu.dk (S.A.M.); aleb@mmmi.sdu.dk (A.E.); ukwiil@mmmi.sdu.dk (U.K.W.)

**Keywords:** health informatics, blockchain technology, Web 3.0, decentralized applications

## Abstract

Background: Healthcare information systems are hindered by delayed data sharing, privacy breaches, and lack of patient control over data. The growing need for secure, privacy-preserved access control interoperable in health informatics technology (HIT) systems appeals to solutions such as Blockchain (BC), which offers a decentralized, transparent, and immutable ledger architecture. However, its current adoption remains limited to conceptual or proofs-of-concept (PoCs), often relying on simulated datasets rather than validated real-world data or scenarios, necessitating further research into its pragmatic applications and their benchmarking. Objective: This systematic literature review (SLR) aims to analyze BC-based healthcare implementations by benchmarking peer-reviewed studies and turning PoCs or production insights into real-world applications and their evaluation metrics. Unlike prior SLRs focusing on proposed or conceptual models, simulations, or limited-scale deployments, this review focuses on validating practical BC real-world applications in healthcare settings beyond conceptual studies and PoCs. Methods: Adhering to PRISMA-2020 guidelines, we systematically searched five major databases (Scopus, Web of Science, PubMed, IEEE Xplore, and ScienceDirect) for high-precision relevant studies using MeSH terms related to BC in healthcare. The designed review protocol was registered with OSF, ensuring transparency in the review process, including study screening by independent reviewers, eligibility, quality assessment, and data extraction and synthesis. Results: In total, 82 original studies fully met the eligibility criteria and narratively reported BC-based healthcare implementations with validated evaluation outcomes. These studies highlight the current challenges addressed by BC in healthcare settings, providing both qualitative and quantitative data synthesis on its effectiveness. Conclusions: BC-based healthcare implementations show both qualitative and quantitative effectiveness, with advancements in areas such as drug traceability (up to 100%) and fraud prevention (95% reduction). We also discussed the recent challenges of focusing more attention in this area, along with a discussion on the mythological consideration of our own work. Our future research should focus on addressing scalability, privacy-preservation, security, integration, and ethical frameworks for widespread BC adoption for data-driven healthcare.

## 1. Introduction

Health informatics technology (HIT) is constantly evolving towards a data-driven paradigm, and the global healthcare IT market is projected to reach around USD 880 billion by 2030 [1]. With its transformative advent to improve patient care and participation, HIT is actively being examined for its ability to address healthcare challenges such as security, privacy leakages, interoperability, and governance in healthcare data management [2]. The root cause of these challenges is broadly observed by researchers as the lack of interoperability between disparate health information exchange (HIE), which is inherently triggered by the lack of seamless communication of electronic health records (EHR), and the proliferation of incompatible data systems [3]. The National Health Service (NHS) UK initially aimed to digitize all patient EHRs by 2018, but faced delays due to disjointed HIEs, a lack of semantic interoperability, a lack of digital skills of the workforce, and a lack of funding, delaying this initiative to 2020, then 2023, and now more assuming 2026 [4]. Furthermore, with the arrival of personalized medicine, genomic data, and remote patient monitoring and care, secure data sharing becomes paramount to achieve secure data sharing, drug tracking, verification, and traceability across the entire pharma supply chain process to combat drug abuse, counterfeiting, and fraud detection [5]. Blockchain (BC) is recommended as a distributed ledger technology to address critical HIE issues using novel design and development methods that ensure efficient patient data sharing of medical records by adhering to patient data privacy, security, privacy preservation, access control, and supply chain tracking, as well as genomic data profiling, credentialing as per compliance laws [6,7].

BC is an immutable decentralized ledger managed by a network of consensus-based nodes that store temper-resistant data to secure exchange without the involvement of any central authority [8]. It adds the trust layer using cryptographic techniques, advanced encryption signatures, and consensus algorithms for the storage, exchange, registration, and management of healthcare data [9]. In addition, the inherent capabilities of BC, such as decentralization, access control, and preservation of privacy, are more focused on research in healthcare components to exchange and disseminate massive amounts of patient consensus data [10]. However, significant BC development, including a wide range of usability tests, proof-of-concepts, and decentralized applications (DAPPs), revolutionized many in the contexts of real-world healthcare interventions in stages [6]. In the first stage, BC applications focused on digital currencies as a peer-to-peer cryptocurrency development. The second stage then shifted towards developing smart contracts for the real estate sector by automating their notarized process and finance sector. The third stage of BC involved non-financial sectors, including academia, government, culture, and healthcare [11]. Our research observed that Blockchain 4.0, with its prominent features of data immutability, decentralization, and access control, enabled practical transformation and invoked artificial intelligence (AI) and machine learning (ML) as value bridges to provide more powerful interventions for healthcare 4.0. This also proclaimed BC’s pragmatic shift in the way of new novel frameworks, development methods, and standard evaluation metrics to develop trusted HIE systems. The promise of a trusted decentralized database for health information exchange (HIE) is a key insight, as it allows comprehensive access to a patient’s entire medical history in a manner that is secure, efficient, and fairly controlled when it comes to whom and when to give and take. In addition, the immutability of BC records ensures data integrity, which is vital to prevent issues such as drug counterfeiting, abuse, and medical fraud. Finally, BC also has the capability to reduce possible human errors by fixing accountability using the hash log of the nodes, which is kept in such a way that every node has a hash of the previous node.

Although such optimistic implications are extended by BC for economic, public, and business transformation, it still appears to be in its infancy regardless of their strengths contributing to healthcare immensely. According to recent research, due to the widespread perception of return on investment (ROI) in BC, it is likely that businesses are considering a vigilantly pragmatic approach and perspective on public health expenditure and policies and helping the government invest substantial capital in the future for the transition of their business processes based on BC technology and Web3 [12]. This means that BC had to realistically show interventional provenance and quantitative measures to meet industry optimism by overcoming technical and regulatory barriers [13]. Both health workers (doctors) and end users (patients) are not well versed in the way BC works; the lack of technical skills for decentralized data processing is another prominent hindrance in maturing the implementation of BC [14]. Researchers are spending reasonable time examining the implementation and uses of evolving BC, which is expected to radically change healthcare business transitions. However, keeping organizational barriers and the lack of technical knowledge and governance challenges in mind, users and stakeholders are not well versed in adopting to BC evolving and its qualitative and quantitative strengths for HIT.

Moreover, BC is not without limitations. Technical concerns, such as high energy consumption, limited transaction throughput, latency issues, and scalability bottlenecks, pose significant barriers to its broader adoption in healthcare, an industry that requires both speed and reliability. These trade-offs must be acknowledged and understood by knowing the strengths provided by the implemented solution intended to evaluate its practical utility in healthcare. Therefore, this lack of comprehension or confusion in current knowledge made us interested in performing an SLR on the examination of the uses of BC healthcare implementations, which fully meet the expectations of the HIT system. In doing so, our included studies aim to provide both qualitative recommendations and quantitative outcomes to support its adoption, often assisted by rigorous validation to ensure practical relevance and provenance. To the best of our knowledge, the majority of current literature and research (Table 1) remains focused on conceptual frameworks or proof-of-concept (PoC) models. Although these early-stage prototypes are valuable for ideation and hypothesis testing, they fail to offer comprehensive insight into practical BC limitations in the real world, such as scalability, instability, and performance. Specifically, PoCs often operate in controlled or simulated environments, lack integration with legacy health systems, and do not adequately address operational challenges such as compliance, stakeholder adoption, and infrastructure constraints. Therefore, they are insufficient for meaningful benchmarking of BC efficacy in clinical and administrative settings. While many of the BC-based implementations reviewed demonstrated potential in clinical settings despite most of them being in the proof-of-concept stage, few have been fully integrated into clinical production environments.

**Table 1:** Previous related SLRs: yes/no.

Ref:	Authors, Year	Research Questions/Objectives	Followed Systematic Methodology i.e., PRISMA Guidelines	Reviewing Answers Similar or Close to Proposed Questions	Identified Strengths and Weaknesses	Identified/Reported Interventions or Developments	Bias or Quality Assessment
[15]	Emeka Chukwu et al, 2020	Yes	Yes	No	No	Yes	No
[16]	R. Saranya et al., 2023	No	No	No	No	No	No
[17]	Reval Prabhu et al., 2023	Yes	Yes	No	No	No	No
[18]	Shashank Chauhan et al., 2022	No	No	No	Yes	No	No
[19]	Shweta Mittal et al., 2024	No	No	No	No	No	No
[20]	Hanaa Fatoum et al., 2021	No	No	No	No	No	No
[21]	Saumya Upadhyay et al., 2024	No	Yes	No	Yes	Yes	No
[22]	Sarthak Dhingra et al., 2024	Yes	No	No	Yes	Yes	No
[23]	Kianoush Kiania et al., 2023	Yes	No	No	No	No	No
[24]	M. Kassab et al.,2021	Yes	No	No	Yes	Yes	Yes
[25]	Shubhangi V Urkude et al., 2023	No	Yes	No	No	No	No
[26]	Ammara Karim Noon et al., 2021	Yes	No	No	No	Yes	No
[27]	Vazirani et al.,2019	Yes	Yes	No	No	Yes	Yes
[28]	Huma Saeed et al., 2022	Yes	Yes	No	No	Yes	No
[14]	Elangovan et al., 2022	No	Yes	No	Yes	Yes	Yes
[29]	Valeria Merlo et al., 2023	No	Yes	No	Yes	Yes	No
[30]	Sobiya Arsheen et al., 2021.	No	No	No	No	No	No
[31]	Alaa, Haddad et al., 2022	Yes	Yes	No	Yes	Yes	No
	**Proposed SLR**	**Yes**	**Yes**	**Yes**	**Yes**	**Yes**	**Yes**

Perhaps most of the conducted SLRs are also very old. Consequently, this review uniquely narrows its focus to studies evaluating real-world BC implementations in healthcare. By analyzing BC healthcare deployments using our systematically validated review protocol followed by PRISMA statement guidelines, this work aims to uncover the strengths, operational challenges, and limitations of BC technology when applied in the real healthcare context, offering a grounded and pragmatic contribution.

Table 1 illustrates that none of the previous systematic literature reviews (SLRs) conducted on BC implementations in healthcare published during the last five years could answer all of our research questions given below in Table 2. Hence, we want to obtain current pragmatic knowledge for healthcare stakeholders and researchers on BC-based healthcare implementation, their design and development methods, strengths and weaknesses, and outcome evaluations.

The review of related SLRs shows how the authors approached the topic differently. For instance, while some prior reviews [13,27] provide useful theoretical insights, they often lack engagement with real-world implementations and pragmatic evidence. These reviews typically focus on proposing BC’s potential without thoroughly assessing practical challenges, such as integration with existing healthcare infrastructures, methodological rigor, and technical barriers to BC, including scalability, latency, and transaction throughput. Other examples, such as two of these [14,28], dealt with information of obvious interest to us, but their methodology focuses more on reporting than validating pragmatic evaluations. Their methodology summarizes past and present trends in academic research topics in this field and discusses healthcare domains where BC could benefit by showing a few proposed implementations.

Furthermore, we reviewed the methods of other previous SLRs [14,23,26] and observed the reporting of an overview of 22 BC-based healthcare studies and their characteristics, but with a detailed review of their operational behavior, development design and methods, and their implementation challenges while adopting BC found missing. This shows that many reviews overlook the complexities of scaling BC technologies within the healthcare ecosystem, such as ethical and regulatory compliance, interoperability with legacy systems, and real-world usability. This review aims to fill this gap by offering a detailed evaluation of practical BC applications and evaluating their effectiveness in real-world healthcare settings.

However, as we summarized, related reviews inadequately provide useful pragmatic knowledge and often emphasize theoretical potential validations without tangible evidence of evaluation effectiveness, leading to actual deployment challenges or measurable outcomes. Therefore, this SLR can systematically focus on actual BC-based implementations that synthesize how these challenges were addressed, evaluate solutions, and provide actionable design, development, and insight for future projects. So, our planned set of research questions and associated rationale aims to identify, report, and contribute as follows:

1. Identify and describe recent (past 5 years) research trends on how (the use of) BC technology reshaped healthcare applications.
2. Explain existing healthcare systems where BC applications are practically implemented to achieve an appropriate solution to real-world problems or digital transformation.
3. Report on BC-based design and development methods applied for implementation which demonstrated the robustness and validity to achieve efficient results for healthcare applications.
4. To elaborate on the strengths and weaknesses that enable or limit various BC-based healthcare applications and interventions based on evidence strengths.
5. Report both qualitative and quantitative data for BC-based implementations, their data analysis or research methods, healthcare datasets, and their evaluation metrics that show validated outcomes to present and lead future research prospects in BC-based healthcare applications.

The rest structure of this SLR is set up as follows: In Section 2, we elaborate an explanation of the review protocol and follow the PRISMA 2020 statement as a research methodology to search, evaluate, and select the literature. In Section 3, we present results by answering all of our formulated research questions. However, Section 4 discusses and elaborates a summary of the findings of all eligible studies that were practically implemented with their indicating that research direction, needs, and pertinent contributions have been carried out on the subject of healthcare utilizing BC technology. Then, Section 5 illustrates the observed challenges and future prospects with research directions. Finally, Section 5 wraps up this SLR and explicitly discusses key takeaways from selected studies for pragmatic evaluation.

## 2. Methods

This section uncovers our review protocol to carry out this SLR formulated in accordance with the 2020 statements of preferred reporting elements for systematic reviews and meta-analyses (PRISMA) [32] and its extension PRISMA-P [33] and PRISMA-S [34], where P stands for ‘protocol’ and S is ‘search’. All authors follow the comprehensive step-by-step guidelines in stages to ensure transparency, precision, consistency, quality, and evidence trustworthiness through quality assessment.

Therefore, the PRISMA checklist has been followed as given in Supplementary File S3, and the protocol is registered in the Open Science framework (OSF) registries by making it publicly available at DOI (https://doi.org/10.17605/OSF.IO/Q3SRF). The steps of the protocol are given later in this section to provide a series of instructions to achieve the answers with the research questions (Table 2) and the attached rationale.

### 2.1. Search Strategy

For a comprehensive search, we followed PRISMA-S [33] due to the high precision of our literature. We conceptualized and explored the related literature according to search components to focus on the development of this systematic review. It offers extensive guidance tailored to different types of information sources and methods. We emphasized documenting all elements of the search process, which aids in making reporting easier and more comprehensive by identifying the number of records of search machines that address our focused research questions related to BC for healthcare in its avenues, development, and implementations, including their strengths and weaknesses. At the beginning, we used the keywords ‘blockchain’ OR ‘distributed ledger’ AND ‘healthcare’, then queries with mesh terms were formulated with the consultation of expert librarians from the University of Southern Denmark. These advanced search queries were mutually formulated to prompt the search literature of the last five years (July 2019 to July 2024) using five search machines: ScienceDirect, IEEEXplore, PubMed, Web of Science, and Scopus. This means a different combination of key terms was run to find the most relevant literature for selection or studies machine-by-machine (Supplementary File S1).

### 2.2. Selection of Studies

After fetching the records from five search machines using advanced string-wise queries, we exported them into RIS files to import them back into the Covidence tool. This tool automatically removed all duplicates in the first instance; then, two independent reviewers initially screened the key terms ‘blockchain’ and ‘healthcare’ from the title and abstract screening in the first review stage. Double-checking the title and abstract was also conducted as a second round. In the second stage, two reviewers (MS AND SAM) evaluated the full-text review of the screened studies following our predefined eligibility criteria (Table 3) to select the studies, whether included according to the given inclusion criteria or excluded assigned for reasons of exception. The conflicts at this stage were resolved by a third reviewer, i.e., the referee (AE), to solve them with the consensus of the reviewers.

We include English language studies as IC2, which may introduce a potential language bias. Non-English studies were excluded due to practical constraints, such as resource limitations and a lack of knowledge of a comprehensive multilingual review process by reviewers. We acknowledge that this exclusion may limit the diversity of included studies, particularly those that offer valuable insights from regions where English is not the primary language. So, we reported this potential language bias as a limitation too. However, the screening procedures for this systematic review have been carefully designed to balance practical considerations. Two screening rounds, blinding procedures, clearly predefined inclusion/exclusion criteria, independent screening, and systematic reconciliation processes are all intended to enhance the reliability and validity of the review findings. By adhering to these rigorous procedures, we aim to provide a thorough and unbiased synthesis to assess the strength of the evidence and facts regarding BC technology’s role in healthcare.

### 2.3. Bias/Quality Assessment and Critical Appraisal

To assess quality critique, this (SLR) intends to examine perceptions or theoretical aspects of BC in healthcare. The Joanna Briggs Institute (JBI) Critical Appraisal Checklist for Qualitative Research was found to be highly relevant since most of the studies are qualitative research. The JBI checklist evaluates the methodological rigor of studies based on various criteria, including clarity of objectives, study design, risk of bias, literature incongruence, and methodological transparency [35]. All 82 studies were assessed with the checklist for critical appraisal by two researchers (MS and SAM), and the results were categorized in the checklist into three groups: high-quality, medium-quality, and low-quality studies. High marked are those that scored predominantly ‘Yes’, medium studies have a balanced mix of ‘Yes’ and ‘Unclear’, and low studies have more ’No’ responses. The specific results, checks, and study quality for each study, including how many studies scored “High”, “Medium”, or “Low” on each checklist item, are summarized in Supplementary File S2. None of the studies were excluded here because only a few studies had any of the low-quality deficiencies, and if so, only on one item with No and Unclear.

### 2.4. Data Extraction and Synthesis

Each study found after the quality assessment was saved in Endnote 21 [36]. Its deduplication option was used to eliminate the left duplicates, if any. All studies were found to be written in the English language. The metadata of all the studies is automatically retrieved by Endnote to double-check and screen for reviewers to avoid any disagreement before extraction. Then, two researchers (MS and SAM) independently reviewed all the studies; if there were any conflicts, the final referee (AE) was present to settle them. Here, extractors observed that the application and developments of BC in healthcare remain as novel methods and included articles with sufficient numerical data, which prevented us from doing the meta-analysis. Therefore, our SLR intends to extract, report, and synthesize data narratively. The extractors reviewed the full text of studies and extracted data onto a shared overleaf full-form customized table having the fields Study Ref, healthcare avenue, problem, solution, Blockchain network, Blockchain type, consensus mechanism, cryptography, encryption, dataset, evaluation metrics, study design methodology, outcome summary, and challenges or limitations. Later on, we observed that studies cover 82 diverse areas or focus on different subfields, and full-form tables were observed as massive to read, so we explored a thematic or topical categorization method to idealize and group studies initially by research questions, then formulated small tables for correlated topics addressed in healthcare avenues, BC design and development method, data analysis techniques, and evaluation metrics. This approach helped us to report the facts and evidence in both a qualitative and quantitative manner.

## 3. Results

**Figure 1:**
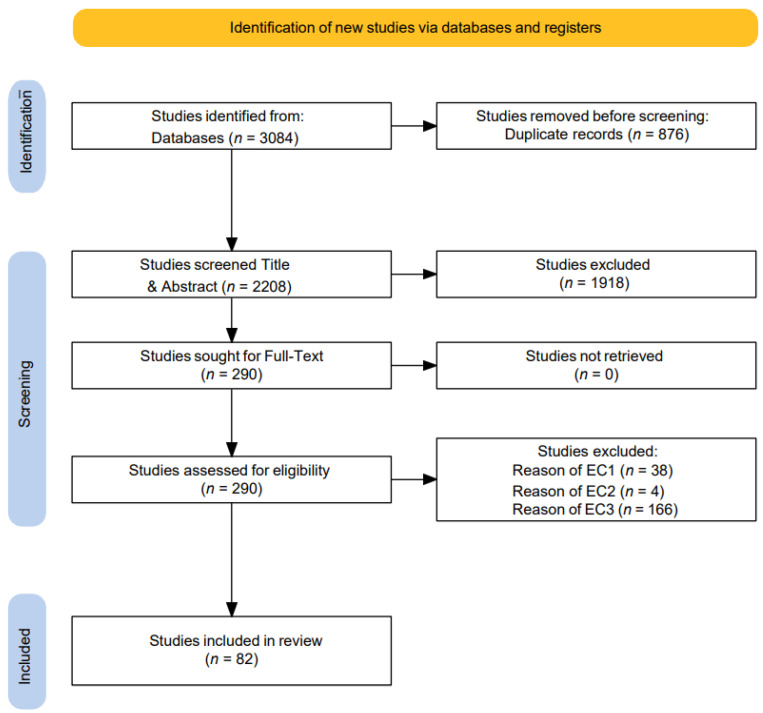
Prisma flow diagram.

This section systematically elaborates on the answers to the research questions (RQs), the interventional outcomes, and their results. In total, 82 primary research studies were included to explain the outcomes of each RQ after assessing their quality and biases, including incongruence with the literature or sources logically defended as potential threats to validity in this SLR. None of the studies were excluded after the study critique, leading only to low bias and precision. However, we made substantial efforts to find all eligible peer-reviewed research articles and conference proceedings from well-reputed search engines, as well as to contact experts in the BC area through LinkedIn. We believe that our research significantly advances the application of BC technology in healthcare interventions. In total, 3084 studies were identified in the original records of five search engines that led to our advanced search queries. After importing them into evidence, 876 studies were identified as duplicates and removed. In the second phase, a total of 2208 studies were screened as titles and abstracts, and 8 duplicate studies were manually identified. During this phase, 1910 were excluded on the basis of post title, abstract, and record screening, as they did not fit with the objectives and rationale of this SLR (i.e., study types and content). The studies were stored in Endnote 21 for the review of the two authors, with the third reviewer present for any disagreements. In the third phase, 290 studies were sought for full text review and 208 studies were excluded and found to be ineligible because they met with exceptions of exclusion criteria. Finally, 82 studies were included as eligible studies for data extraction and synthesis for this SLR that adhered to the research objectives of this study and fully met the inclusion criteria. The PRISMA flow chart is attached in Figure 1.

**RQ1: What is the current state of BC technology research trends in healthcare?**

**Figure 2:**
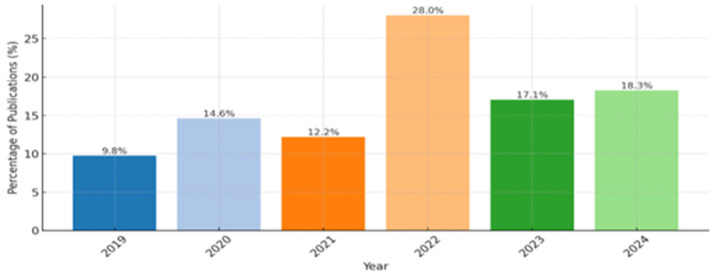
Annual research publications (2019–2024).

**Figure 3:**
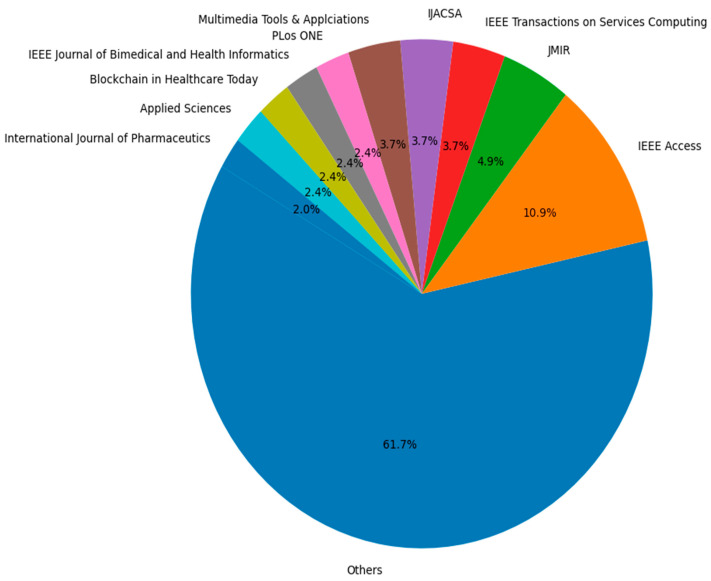
Distribution of research investigations across leading journals.

**Figure 4:**
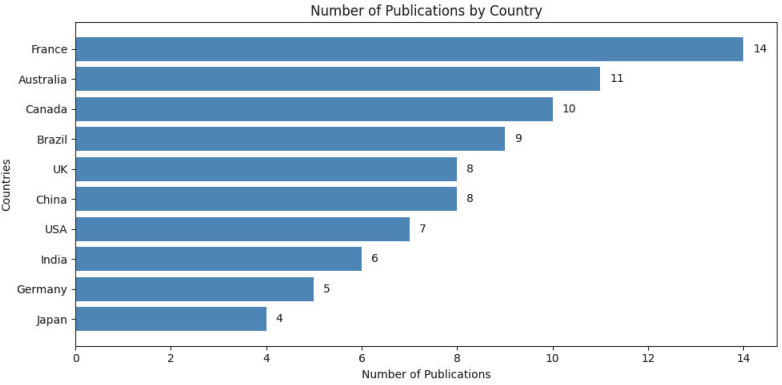
Analysis of research publication output by country.

Here, we obtained descriptive records on the number of articles published annually, their distribution in leading journals, and publication percentages, including analysis of the output by country answered by this SLR (shown in Figure 2, Figure 3 and Figure 4). To complete this SLR, we reviewed previous systematic literature reviews, multivocal reviews, comprehensive reviews, and original research articles published in BC in healthcare between 2019 and 2024. Figure 2 completes the annual distribution of research publications as a percentage of data, revealing a clear upward trend in publication activity, reaching its peak in 2022 at 28% of total publications. The years 2023 and 2024 maintain elevated output levels, with 17.1% and 18.3%, respectively, signaling sustained research engagement. Earlier years show more modest contributions, with 9.8% in 2019 and a gradual increase to 14.6% in 2020. The elative dip to 12.2% in 2021 may reflect external factors affecting publication output.

Similarly, Figure 3 visualizes the distribution of research investigations published in various journals, focusing on the top 10 journals while aggregating the rest as “Others”. The “Others” category comprises the majority, with 61.7% of publications, suggesting a wide diversity of journals contributing to the field. Among the top individual journals, IEEE Access holds the largest share at around 11%, Journal of Medical Internet Research (JMIR) follows with 4.9%, while IEEE Transactions on Services Computing and International Journal of Advanced Computer Science and Applications each contribute 3.7%. The remaining journals, including Multimedia Tools and Applications, PLOS ONE, and others, contribute between 2.4% and 3.7% each. This output reflects both the concentration of research in specific high-impact journals and a broad spread across numerous other outlets, highlighting the multidisciplinary nature and reach of research in this field.

However, Figure 4 distributes the authors and their institutional affiliation of the studies in 10 countries. Six countries, France (*n* = 14), Australia (*n* = 11), Canada (*n* = 10), Brazil (*n* = 9), UK (*n* = 8), and China (*n* = 8) cumulatively represented 65% of the publication contribution.

The United States, India, Germany, and Japan show comparatively lower publication counts, with Germany and Japan at the bottom with five and four publications, respectively. This distribution underscores the diverse research capacities across regions, with European and North American countries generally displaying higher publication volumes, suggesting varied levels of emphasis on research output and resources allocated to scholarly work in these nations.

**RQ2: Which fundamental healthcare avenues used BC technology to solve the issues raised in earlier studies?**

In this section, we will primarily explain the foundation of BC and how it deals with prominent healthcare challenges noted by earlier researchers. In addition, we will discuss potential healthcare avenues and their pragmatic applications leveraged by BC technology that has provenance at the level of development. BC is a distributed, decentralized digital ledger technology intended to log transactions safely over a network of computers. Using cryptographic techniques guarantees data confidentiality and integrity, eliminating the requirement for a central authority to supervise or validate the system. Transparency and trust are ensured since once data are stored in a BC, it is practically difficult to change them later without the consent of the network [37]. Simply put, our extensive literature review taught us that BC enables the ultimate development of a decentralized, trusted, accurate, verifiable, immutable, consensus-encrypted data ledger, and secure communication network without intermediary.

BC’s adaptability was initiated from cryptocurrencies but rapidly expanded in areas such as real estate, supply chain, gaming, finance, and healthcare, driven by its ability to create trust and immutability in decentralized systems [15]. This makes BC particularly appropriate for tasks where data systems are disjoint and scattered and records exist in silos, which compromises or even misleads the secure exchange of health data. Since health data are crucial, HIT researchers are making reasonable efforts to bring transformations through BC. An active illustration of data integrity, efficient accessibility, secure data sharing, and immutability in healthcare is HealthChain [38], which is an infrastructure built on this technology that provides a chain of custody to the database. In addition to this, many other BC implementations in healthcare solve the challenges that must be addressed for its widespread adoption. According to [37,39,40], scalability is a major challenge because healthcare systems produce huge volumes of data, and existing BC platforms frequently find it difficult to manage large transaction volumes and large datasets effectively. Another difficulty is integrating BC with current systems; many healthcare organizations still use outdated systems, making it difficult to do so without investing in expensive updates. Legal and regulatory compliance is very complicated, especially when it comes to following data privacy regulations such as GDPR and HIPAA, which demand strict protections for private patient data. Public BCs reveal metadata even when content is encrypted, raising concerns about patient confidentiality and data privacy. Implementing and maintaining BC systems can be costly and resource intensive, requiring specialist knowledge that many healthcare companies lack. Lastly, cost and technical incompetence, such as throughput, latency, usability, and resource consumption, are considerable obstacles [28].

**Figure 5:**
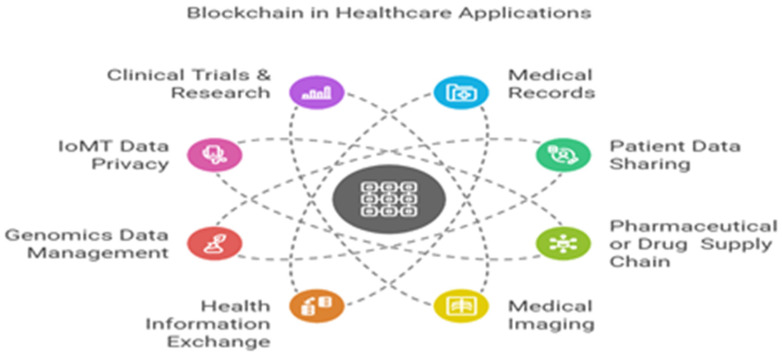
Healthcare avenues leveraging BC technology.

Therefore, our research on BC-based applications in major areas of healthcare covers important topics. It demonstrates how these systems could undergo radical change and address the mentioned challenges as a matter of urgent need. As shown in Figure 5, we explored numerous multi-purpose BC-based applications that are developed in the following major healthcare avenues:

- **Medical records:** BC is widely leveraged in electronic, medical, and personal health records (EHR, EMR, and PHR) implementation, integration, and management to enable access control, privacy, high security, and seamless and timely sharing of the sophisticated volumes of patient data. Of 82 included studies, 39 (31.98%) studies were found to have been implemented and published on this avenue to facilitate prominence problems and likewise fragmented EHRs, security risks, data inaccessibility, unauthorized access, and data leakage and even loss [31]. The EHR, EMR, and PHR are digital organizations that collect, manage, and store patients’ medical, personal, and health information, providing a comprehensive and holistic view of their healthcare journey. They are the cornerstone for modern healthcare systems because they integrate diverse datasets, enhance care coordination, and enable data-driven decision-making. Their management also becomes paramount while dealing with fragmented records and unlawful access. BC ensures secure patient-centric control over records by utilizing framework implementations such as Hyperledger Fabric and Ethereum, which improve data accessibility, privacy, and interoperability [41,42,43].
- **Patient data sharing:** In patient data sharing, BC tackled the issue of inefficient and insecure sharing mechanisms. Traditional systems often fail to provide patients with control over their data, leading to privacy concerns. BC-enabled solutions such as HealthChain [38] utilize proxy re-encryption and smart contracts to allow patients to dynamically share their data with specific stakeholders (e.g., providers, researchers) while maintaining privacy. This approach has shown improved efficiency and transparency, particularly in multi-institutional data exchanges [38,44,45]. Future applications include integrating AI with BC for personalized and secure patient care analytics.
- **Pharmaceutical or drug supply chains:** The pharmaceutical supply chain benefited significantly from BC’s ability to ensure transparency and traceability. Counterfeit drugs pose a major risk to public health, and BC systems such as Hyperledger Fabric for the drug supply chain address this issue by creating immutable records of drug transactions. One study showed a 100% success rate in counterfeit detection and a 95% reduction in supply chain fraud. Additionally, compliance with regulatory standards such as DSCSA is streamlined through BC’s auditable and tamper-proof nature [46,47,48].
- **Medical imaging:** BC introduced decentralized solutions for medical imaging, addressing privacy concerns and the high storage costs associated with large imaging files such as MRIs and CT scans. The patient-centric medical image management study used Ethereum and inter-planetary file systems to store medical images securely and cost-effectively. This decentralized approach ensures that imaging data are accessible only to authorized users, enhancing patient trust and reducing reliance on centralized repositories [49,50].
- **Health information exchange:** Interoperability and data ownership are significant challenges in health information exchange (HIE). BC solutions compliant with standards such as HL7 FHIR enable secure and seamless data exchange between healthcare institutions. The patient-centric health information exchange framework study demonstrated how BC improves interoperability while empowering patients with control over their data. This system significantly reduces redundancies and enhances the scalability of large-scale health information systems up to large transactions [51]. An Ethereum-based implementation of health data exchange [52] showed their chain effectiveness, namely EdgeMediChain, evaluated for execution time with a reduction of nearly 84.75% for 2000 concurrent transactions, claiming higher throughput compared to a traditional BC and scalable ledger storage with a linear growth rate implemented by other BC-based frameworks [53]. The authors implemented a smart contract for patient-centered data control using public key cryptography on simulated data, providing enhanced patient data ownership, scalability, and security.
- **Genomics data management:** Given the sensitivity of genomic information, privacy and ownership are critical concerns in genomics data management. BC applications, such as non-fungible token (NFT)-based systems for genomic data sharing, allow for privacy-preserving collaboration in genomic research and mutation of diseases. The BC for genomic data auditing study showcased how immutable audit trails enable researchers to securely query genomic datasets while ensuring compliance with data ownership policies [54]. It additionally [55] demonstrated that the evolution of genomic logs ledger via the platform (MultiChain 1 to 2) returned a 30–40% increase in insertion logs efficiency, showing a creative and efficient technique for storing and querying genomic log file data.
- **Internet of Medical Things (IoMT) data privacy:** The IoMT devices and remote networks are major contributors that generate massive amounts of real-time data from sensors and wearable devices, and nowadays, these are on rapid adoption and deployment [56]. They are mostly used for remote patient health monitoring by a healthcare provider, and this mode raises concerns about data security and privacy. BC frameworks integrated with lightweight cryptographic protocols addressed these challenges by enabling secure data sharing in IoMT environments. The IoMT-enabled EHR privacy management [57] demonstrated real-time data protection with reduced latency and high throughput, making BC a viable solution for IoT-heavy healthcare systems, while [58] proposed a multiple authority BC-based attribute-based encryption EHR access control scheme to solve the data security and access control for IoMTs environment through four smart contracts.
- **Clinical trials research:** BC has the potential to enhance the accountability, audibility, and transparency of medical researchers and practitioners in the areas of managing trials, subject consents, and clinical trials themselves. By preserving the immutable logs of patient consent data, authorities could readily regulate the quality of clinical trials, ensuring that it corresponds with the citizen’s law and complies with an informed agreement. It is particularly crucial to save from many prevalent forms of clinical fraud, medication abuse, and falsified informed consent forms. It also entails changing records and fabricating patient permission, suggesting that trial subjects’ authentication is crucial to preventing them. Here, BC leverages smart contracts to avoid and even help reinforce that kind of arrangement in a way that prevents physicians from accessing patient data without a key by the conclusion. Therefore, [59] proposes a proof-of-concept protocol that utilizes BC to timestamp every stage of patient consent collecting, archiving, and historicizing the consent through cryptographic validation in a visible and safely unfalsifiable manner. These healthcare avenues demonstrate the wide range of applicability and the transformative potential of BC-leveraged solutions such as decentralized, holistic, predictive (EHR, EMR, and PHR) systems, improving patient data sharing interoperability, drug traceability counterfeiting, and so on. All applications we explored addressed significant pain points extensively to contribute to broader challenges such as large-scale scalability, integration with legacy systems, health data management, preservation of privacy, and cost efficiency. Our research contributes to the growing body of pragmatic evidence supporting BC’s role in enhancing healthcare security, transparency, privacy, interoperability, data management, and patient centricity, but is not limited to this. We summarized some BC-based applications in informed healthcare avenues in Table 4.

**RQ3: What are the strengths and weaknesses raised by existing developed BC-based healthcare applications?**

This section of our research intends to explain the strengths and weaknesses and to form their common aspects by analyzing the 82 included studies in a systematic manner to synthesize their objective contributions, assess their findings, and evaluate the methods they applied. In doing so, we observed common prominent aspects associated with the objectives and the contribution of various studies, showing an interconnected and correlated nature of the objectives, such as [51], which implemented a patient-centric interoperability framework for HIE. Moreover, other BC implementations also focused on diversified, integrated areas of contribution such as in patient data sharing, HealthChain [38], medshare [60], and in the pharmaceutical supply chains PharmaChain [61] and BRUINchain [62], in clinical trials [63], in IoMT Data privacy [57,58], in HIE-MedChain [64], MedHypChain [65], MEXchange [53], EdgeMediChain [52], BaaS-HIE [66], in genomics data management-POPS-G [54], MultiChain [55], and in medical imaging PCMIMS [49] and PCRIM [67]. During analysis of all these applications, we identified positive outcomes as strengths by evaluating their robustness of methods, showing validated experiments carried out to assess common aspects such as data security privacy, decentralization control, interoperability, etc., commonly set as objectives [68,69]. On the other hand, the extent of unresolved issues [70] naturally leads to weaknesses in the form of the same or close to the aspects such as security imbalance, scalability, technical complexity, and integration with legacy systems, that is, lack of interoperability [71]. So, by understanding these diversified, causal, and common relationships, this work analyzed and collected the identified positive outcomes (strengths) and unresolved issues (weaknesses) in the form of the same observed aspects, which eventually helped researchers and developers to create more innovative solutions, such as Layer 2: scaling, sharding, privacy-enhancing technologies, tokenization, and energy-efficient consensus mechanisms.

**Table 4:** Summary of applications for BC-based healthcare avenues.

Healthcare Avenues	Applications	Problems	Leveraging Blockchain	References
Medical records (EHRs, EMRs and PHRs)	Privacy-preservation EHRs/EMRs	Fragmented EHRs, security risks, data inaccessibility, incomplete view, data leakage, and unauthorized access.	Enhances data accessibility, privacy, access control, and provides a holistic view of patient history.	[42,44,68,69,72,73,74]
	Patient-centric EHRs/EMRs/PHR	Data loss, lack of patient control due to centralized EMRs, tempering, communication gaps among hospitals, and inefficient clinical data retrieval systems for patients.	Provides data immutability and integrity through various conventional consensus algorithms and hash functions, and also enables decentralized control over electronic medical records (EMRs).	[41,43,75,76,77,78,79,80,81,82,83,84,85]
	Interoperability of EHR/EMR	Cross-chain interoperability, application-level interoperability, and delays in seamless data exchange. Lack of standardization.	Distributed and decentralized translation layer and integrating HL7 FHIR resources, achieved application and semantic, cross-chain interoperability solution allowing BC networks to inter-communicate, share data, and make requests.	[50,86,87,88,89,90,91,92,93,94]
	Patient consent, access control and full- patient control EHRs	Patients’ intractability led to difficult communication and increased procedural costs, limits in developing a better understanding and consent-based patient’ traceability of their data. Medical records theft due to the centralized system. Personal data leakages and monetization.	Enables improved cryptographic primitive multiple access control, attribute-based and semi-policy hiding, and dynamic permission changing based on partial ciphertext simultaneously.	[47,95,96,97,98,99,100]
	BINDAAS: BC-based deep-learning as a service	Privacy, scalability, and predictive challenges in EHR data	Secure and predictive EHR management using BC.	[101]
Patient data sharing	HealthChain	Information security, interoperability, data integrity, identity validation, and scalability.	Presents patients and providers with access to consistent and comprehensive medical records.	[38]
	System for the promotion of traceability and ownership of health data	Privacy risks and inefficiency in data sharing.	Secures access and privacy in patient data sharing with BC technology.	[102]
	MedShare	information isolation, centralized health servers, distributed denial of service (DDoS) attacks, and the single point of failure.	Efficient patient data sharing with fine-grained access control and constant size attribute-based encryption (ABE) scheme.	[60]
	Redact-chain for health	Data modification and deletion on BC and centralized patient systems undermines single point of failure and robustness.	Redact-Chain integrated with distributed trapdoor scheme introduces a fine-grained data editing scheme for institutions to effectively edit and manage data on the BC, thereby circumventing the issue of a single point of failure.	[103]
Pharmaceutical or drug supply chain	Model for storing pharmaceutical supply chain data	Trust, relying on third party, and storing large data.	Completeness, cost-effectiveness, privacy, and confidentiality ensured and efficient information queries.	[46]
	Medledger	Counterfeit drugs having serious side effects, online, Internet-based pharmacies hazards safety and security.	Provides efficient and safe transaction records, with high integrity, reliability, and security, and reduces the likelihood of meddling with stored data.	[104]
	Counterfeit drug prevention pharma supply chain, PharmaChain and BRUINchain	Counterfeit fake drugs and lack of drug traceability.	Provides traceability, tracking, verification and effectively forestalls any medication being scanned, expiration detection, fake and counterfeit detection; as with reduced paperwork.	[61,62,105]
	Healthcare chain network	Prescription drug abuse and doctor shopping.	Tracking prescriptions by decentralization and audibility, encryption ensured anti-fraud and anti-forgery measures to prevent prescription abuse.	[106]
	Secure pharmaceutical supply chain	Time delays in the supply chain and lack of security.	Timestamping, authentication, process coordination, non-repudiation, commercial transactions, and security for transactions and storage.	[107]
Medical imaging	PCMIMS—patient-centric medical image management system, and PCRIM—patient-centric test report and image management system	High operational costs and storage space requirements, security, and privacy of large medical images.	Efficient validated large patient-centric medical image processing and requesting, decentralized storage, and privacy of medical imaging data.	[49,67]
Health information exchange (HIE)	Patient-centric health information exchange framework	Data ownership issues, interoperability challenges.	Facilitates interoperability and ownership of patient data.	[51],
	MedChain	Trustworthiness of a third-party, inefficient sharing data streams from IoT and sensors.	Higher efficiency and satisfy the security requirements in data sharing efficient scheme.	[64]
	MedHypChain	Infected patients overwhelmed the inadequate medical services and lack of interoperability.	Achieved confidentiality, anonymity, traceability, and unforgeability through an identity-based broadcast group encryption scheme.	[65]
	MEXchange	Security, privacy, and costs, with focus on privacy issues caused by analyzing senders and receivers of transactions in HIE.	Ring signature and stealth address implementation mitigating privacy and security issues among healthcare stakeholders.	[53]
	EdgeMediChain	HIE systems unable to adapt the expanding volume of body sensor data and their vulnerabilities against attacks.	BC and edge computing facilitate providing an integrated healthcare ecosystem in terms of scalability, security, and privacy to execute up to 2000 concurrent transactions.	[52]
	BaaS-HIE	Lagged (EHRs/EMRs) adoption due to confidentiality, interoperability, integrity, and privacy.	Offering decentralized and fine-grained accessibility mechanism for the patient and the doctor in a given healthcare system.	[66]
Genomics data management	Privacy and Ownership Protection System for Genomics [POPS-G]	Privacy and security of gnomic data during analysis and maintenance for mutation of disease.	Provides BC-NFT genomics data handling system for secure, efficient, and simple while preserving privacy and ownership of merging genomic data with EHRs.	[54]
	Genomics audit logging (MultiChain)	Time or space-efficient structure and mechanism of storing/retrieving genomic data access logs on MultiChain	presented creative and efficient techniques for storing and querying genomic log file data in MultiChain.	[55]
IoMT data privacy	MediVault	Security risks associated with the current digital healthcare data, accessing data by malicious parties.	Provide safe storage of healthcare data in digital form, secure access for authenticated entities and secure, simple, and efficient with a limited computation overhead.	[57]
	Multiple authorities attribute-based encryption for EHR access control scheme	Security risks of sharing patients’ EHRs, prevailing problems such as difficulties in sharing EHRs between different hospitals and patients’ inability to grasp the usage of their medical devices.	Ensures trust of multiple authorities, secret sharing and smart contracts-based keys for multiple administrative attributes for a single point of failure reduces communication computation overhead on user data. Ciphertext encryption ensured the security of EHRs and IoMTs.	[58]
	IoMT-enabled EHR privacy management	Unauthorized access and privacy risks for IoT and wearable device data.	Ensures secure real-time data sharing for wearable and IoT devices using lightweight protocols.	[77]
Clinical trials and research	BC-based patient consent management	Inefficient and non-transparent consent management systems.	Transparent and efficient consent processes for patients.	[108]

Being more domain-specific, the strengths explain the inherent design principles, methods, technological tools, and operational requirements of BC-based applications, so identifying these from included implementation helps stakeholders (hospitals, doctors, patients, regulators, and caregivers) to understand the potential benefits before investing, using, and adopting BC solutions for their operations. However, weaknesses expose BC’s challenges in healthcare environments analyzed from methodological limitations, handling large-scale data, integration to existing systems, to maintaining compliance with stringent healthcare regulations. Table 5 summarizes the key strengths and weaknesses identified from the included BC-based healthcare applications.

**Table 5:** Summary of strengths and weaknesses.

Aspect	Strengths	Weaknesses	References
Data security and privacy	Multi-attribute encryption techniques, consensus mechanisms ensure highly secure storage and data transmission and immutable records to reduce tampering risks.	Privacy and security imbalance, especially compliance of GDPR remains a big challenge.	[38,41,42,70,72,93,109,110]
Transparency and traceability	Immutable records build trust. Transparency through smart contracts for patient consent mechanisms to improve decision-making.	Inefficiency and ethical barriers (e.g., data monetization concerns) invoke complicated prevalent adoption.	[25,28,46,47,48,84,88,108]
Decentralized control	Eliminates single points of failure. Empowers individuals (e.g., patients) with data control.	User adoption is hindered by technical complexity and a lack of understanding among stakeholders.	[41,43,61,74,91,95,101,105]
Interoperability	Integration with HL7 FHIR standards enables seamless data sharing and reduces redundancies.	Integration with legacy systems and IoT devices is challenging.	[41,51,64,67,71,73,77,97]
Efficiency	Smart contracts streamline processes and consent management and data sharing.	High costs (e.g., gas fees in public BC) hinder implementation.	[38,44,45,67,95,96,111]
Regulatory compliance	Redactable BC supports detailed audit trails and GDPR compliance, enhancing transparency.	Variations in regulations and ethical concerns limit cross-border implementations.	[25,28,47,48,70,72,84,88]
Scalability	N/A	High computational and storage demands, along with latency issues, and limited scalability.	[49,50,73,110]
Cryptography	N/A	Proxy re-encryption and attribute-based encryption methods demand high computational resources.	[45,109,112,113]

**RQ4: How do BC-based applications, their design development methods, data analysis, and evaluation metrics correlate with present and possible future prospects for healthcare care development?**

This section of our work is divided into three parts. The first is to explain BC design development methods applied by the BC-based application; the second is what type of data they analyzed, and the last is what metrics they evaluated for effective outcomes. BC-based developments have a big impact on how healthcare is conventionally transformed today with a pragmatic shift and a view to revolutionize the industry soon [15]. Applications extensively ensure immutability, integrity, high-level security and privacy in health records (EHRs, EMRs, PHRs, etc.) by enabling patient data ownership, privacy preservation, access control, patient content, interoperability, and a holistic decentralized view of their medical history [38,41]. As our work explained, the BC-based group of implementations in the major healthcare areas (Table 4), their problem statement, and proposed solutions have been derived by utilizing BC design development methods for implementation. In addition, all our included studies in this SLR comprise a diversified range of proofs-of-concept (PoCs), novel model frameworks, applications, and evaluations focusing on testing the performance, feasibility of their proposed research objectives, contributions, and achieved results. So, it becomes paramount to understand that the BC design development method could be re-adopted for further research. BC design and development methods comprehend a range of novel frameworks, methodologies, preliminaries, and best practices to create robust, evaluated, and efficient systems [66]. Common design and development approaches applied in BC-based applications involve BC type (public, private, and consortium), decentralized storage IPFS (inter-planetary file system), network (Ethereum or Hyperledger Fabric), and encryption techniques [28]. In addition, in preliminary designs, decisions are made about the development of BC-based healthcare applications for candidate smart contracts, which are self-executable agreements used to ensure secure and reliable access to healthcare data [113]. These methods provide a solid foundation to design and develop scalable, temper-proof, robust, and secure BC-based healthcare applications tailored to meet specific needs [41,42]. Our research assessed 82 studies comprising healthcare implementations and their effective evaluation using plenty of BC-based design, development research methods applied on any simulated or real patient or health information exchange or health datasets. The implemented solutions show using measurable quantitative results as evaluation metrics using the Hyperledger Caliper, Ethereum testbed, or other benchmarking tools. Those evaluation metrics, i.e., security, privacy, interoperability, latency, throughput, TPS, etc., or performance analysis visualize respective metrics of domain, illustrating that the proposed applications, frameworks, or schemes analyzed by experiments were effective and viable. Therefore, in this part, our aim is to qualitatively report the variety of Blockchain design, development, and research methods to summarize their prominent aspects adopted by researchers to contribute to present developments, as well as the future BC-based healthcare prospects suggested, as shown in Table 6.

**Table 6:** Summary of applied Blockchain design and development methods for contributions.

Applications	Blockchain Design and Development Methods (Blockchain Type, Network, Storage, Smart Contract, and Encryption)	Contribution to Present Healthcare	Future Prospects	References
Patient-centric EHRs/EMRs/PHR	Permissioned BC, Hyperledger Fabric, multiple smart contracts, IPFS, and asymmetric key cryptography	Enhanced data accessibility, privacy, and interoperability.	Real-time, interoperable global EHR systems integrated with IoMT and body sensors.	[41,43,75]
HealthChain	Ethereum, smart contracts, Proxy Re-encryption (PRE)	Improved consent management and secure sharing.	AI-driven data analytics for personalized healthcare solutions.	[38]
Pharmaceutical or drug supply chain	Permissioned BC Hyperledger Fabric, Smart Contract, IPFS, Digital Signatures encryption	Verified drug traceability, fraud detection, accuracy, and drug counterfeit prevention.	Automated supply chain auditing and global compliance integration.	[46,61,107]
PCMIMS and PCRIM	Public BC, Ethereum, IPFS, Smart Contract Asymmetric Encryption	Decentralized storage reduces costs and enhances access.	AI-enabled imaging analysis integrated with secure BC records.	[49,67]
Patient-centric health information exchange	Mixed-BC Hyperledger Fabric, HL7-FHIR compliance, Smart Contract	Improved interoperability and ownership of patient data with full access to treatment.	Seamless cross-chain healthcare information exchange to deliver high-quality treatment.	[51,114]
POPS-G and MultiChain	MultiChain, Ethereum, NFT Metamask storage, Smart Contract, genetic encryption	Secure sharing and controlled access for sensitive genomic data for desease mutation.	Global genomic research collaboration with robust privacy controls.	[54,55]
MediVault	Ehtereum BC, NFT using (IPFS) and FireBase, Smart Contract, asymmetric encryption, and Multi-Authority Signature Scheme	Secure and efficient real-time data sharing for IoMT and IoT devices.	Expansion to smart city healthcare systems leveraging IoT.	[56,57]
MedShare and Redact-Chain for health	Ethereum and redactable Blockchian, IPFS, multiple smart contracts, searchable attribute encryptions, and SHA-256	Transparent and efficient consent mechanisms with boolean search empower patients’ data-controlled sharing with complete view and data editing.	Universal patient-controlled data platforms with editable exchange.	[60,103]

Lastly, BC also provides a universal protocol for data storage systems, as in the first instance, it connects disparate healthcare systems and enables the seamless sharing of patient data/information between providers in a distributed decentralized manner [64]. This distributed ledger also enables to extracting, processing, and deriving insights from data stored on BC systems using a variety of data analysis methods depending on the type and structure of the data. As in [91], the authors processed the simulated data as real-world trials of the EHR application to assess interoperability of integrating the HL7 FHIR resources frameworks built in BC. Their evaluation assessed other respective key metrics, such as interoperability index, network latency, and data-sharing efficiency, which measure Blockchain’s ability to correspond data formats and protocols across disjointed healthcare entities. In addition to this, BC’s data analysis uses the case to combat counterfeit medicine and offers transparent end-to-end tracking of pharmaceuticals [61]. The research design and experimental setup in the area implemented by [62] using BC-integrated supply chains are implemented and tested under controlled conditions. Here, effectiveness is assessed with a 100% success rate for storage efficiency, traceability completeness, and fraud detection rates with 95%, and allows query performance, highlighting BC’s impact on supply chain transparency. We summarized more studies that involve datasets and data training, analysis, and quantitative measures, as can be seen in Table 7.

**Table 7:** Summary of BC applications, data analysis, and quantitative outcomes.

Blockchain-Based Healthcare Application	Data Analysis Performed	Evaluation Metrics (Quantitative Results)	Data Analysis Technique/Models	Datasets	References
PatientDataChain	Real-world PHRs (1144 trasanction) analyzed for data exchange and scalability.	Privacy: 98% with security compliance; scalability: up to 10 k TPS.	Latency and throughput testing, cryptographic model testing	100 real-world PHR records over 1144 transactions	[94]
BRUINchain	Real-world pharmacy data analyzed for traceability and regulatory compliance.	Traceability:100% success in counterfeit detection; scalability: 2 k TPS.	Traceability analysis using BC logs	Real-world pharmaceutical data	[62]
Structural EHRs standard based on the HL7 FHIR	Real-time FHIR simulation of 3 models.	Gas cost: 1.8 billion gas units, gas transactions: 10,000 concurrent.	Hybrid off-chain and on-chain approach, the second model implements a full on-chain	Synthetic dataset with 10,000 patients’ personal information records	[90]
[POPS-G]	Genomic access logs analyzed for query time and space efficiency.	Query time: ∼200 ms per request; space efficiency: reduced storage by 35%.	Query benchmarking and space utilization analysis	Real-world genomic data	[54,67]
BC for Decentralized Patient Records	Cross-border EHRs evaluated for privacy and security.	Privacy: 96% GDPR compliance; security: no unauthorized access in 10 k tests.	Compliance testing and security event simulation	Real-world international EHR datasets	[73]
Pharmaceuticals Traceability using BC	Real-world drug data analyzed for counterfeit detection and fraud prevention.	Traceability: 100% fraud detection; fraud prevention: 95% reduction in cases.	BC transaction analysis and fraud detection modeling	Real-world pharmaceutical data	[84]
BC Technology in Healthcare Systems utilizing Machine learning Techniques	Machine learning techniques and BC test net analysis.	Model accuracy: 97.236%.	SHA-256 Secure hashing, Naive Bayes classification algorithm	Real health dataset with 81 symptoms list of patients	[115]

To update clearer differentiation between experimental solutions and those already integrated into clinical practice, we observed that PatientDataChain, BRUINchain, and HL7 FHIR-based structural EHR standard are currently in prototype or proof-of-concept stages, aiming to demonstrate BC’s potential in improving healthcare interoperability and patient control over data. However, applications such as BC for Decentralized Patient Records and Pharmaceutical Traceability Using BC are fully deployed and operational in clinical settings, actively improving patient data management and supply chain transparency. However, our research examined whether all are implemented at the performance evaluation level. We observed the diverse nature of the studies included in this SLR, each employing different evaluation metrics and quantifying their results; it is challenging to directly convert all metrics into a single unified framework system due to the applied adoption of different technologies and methods. Although some studies report transactions per second (TPS), gas fees, fraud detection, and privacy compliance with varying units and thresholds, a direct normalization might not adequately capture the unique context or significance of each metric within its respective study. Instead, we present these metrics in their original form, highlighting the strengths and limitations of each approach. This method preserves the integrity of the study findings while ensuring that the comparisons remain transparent and contextually relevant.

## 4. Discussions and Limitations

This SLR aims to fully comprehend how BC-based implementations are being developed in the various healthcare production settings at the evaluation level to synthesize their pragmatic change. Data were extracted from the 82 included studies to compile promising results on the most recent knowledge about how healthcare data and systems are transformed by leveraging BC applications utilizing its robust design and development methods to over healthcare real datasets.

- **Pragmatic evolution:** The analyzed findings show the steady progression of BC-based implementations gained a lot of development focus with new creative ideas that help researchers to initiate multi-sphere practicability. The viability of pragmatic shift is being established and evaluated hereof in terms of qualitative recommendations, providing insights into academic, constructive, and quantitative outcomes as measurable metrics in the following perspectives.
- **Health data management:** Based on our findings, we can say that more academics are paying attention to data management and medical records. The use of BC technology for medical data management has been supported by previous studies [41,43,75,76,77,78,79,80,81,82,83,84,85]. Furthermore, BC can help construct an HIE to manage such EHRs, EMRs, and PHRs [47,95,96] by merging heterogeneous types of data [56,111]. Based on the SLR, we identify three primary enhanced facets of ongoing research in this field.
- **Enhanced data security:** Previous research about data security implications of this technology in healthcare focused on handling the privacy of data by maintaining data access permission. However, the to an advanced implemented the identity-based proxy re-encryption (IB-PRE) algorithm used by [42,109] to share EHRs in a private, secure, and safe manner over the various security risks such as amateur-level attacks and data breaches by hackers, their technique involves a proxy node retrieves requested data from IPFS, which performs re-encryption and returns re-encrypted data to the requester to ensure user privacy and data integrity.
- **Privacy preservation:** Data prevention from unauthorized access with maintaining data confidentiality to guarantee data safety are two other major issues covered in our research on the BC’s role in health data management. Most of the included studies concentrated on stopping unauthorized access [42] and preserved privacy through a framework for EHR management. To accomplish this goal, several studies have been put forth, such as the use of redactable BC [72], federated learning technique [71], ring signature and stealth address [53], and consortium BC [73].
- **Access control:** Multiple studies discussed access control as a key element to health data management which guarantees that sensitive patient data are only available to authorized users or systems while upholding legal requirements and privacy. According to our results, refs [58,95] implemented BC-based multiple attribute-based encryption for access control schemes for EHR for patient data sharing in hospitals among multiple authorized entities and achieved trust among them using secret sharing by deploying smart contracts for attributes. Thus, we also observed a few integrated studies such as [45] implemented BC and an AI-based decentralized access control model to enable secure healthcare interoperability and IoT access control implementation for sharing EHR data [47].
- **Advanced novel patient-centric frameworks:** Our included studies also comprise new novel frameworks, models have been implemented and trained by the novel patient-centric architectural framework [75], and trusted artificial intelligence for patient data sharing [59] affirms the patient-centric design of a decentralized healthcare management systems over traditional EHR-based systems who plagued data loss risks, security, and immutability consensus over patient health records. Their models ensure patients with authentic immutable medical histories with predictive capabilities by adding an additional layer of BC novelty and AI capabilities synergy.
- **Advanced interoperability and cross-chain communication:** Interoperability in healthcare involves the secure, seamless exchange of patient data and information across disparate systems, stakeholders, and applications. Our results show a wider contribution of BC as a decentralized and transparent nature, and offer promising solutions to address interoperability challenges. Consider the HIE system based on FHIR, BigchainDB, and GraphQL approach [97], on-chain and off-chain healthcare data system [70], Redact-Chain for Health [103], new scheme BaaS-HIE [66], Appx-Chain: application-level interoperability [86] enabled health data exchange to use the FHIR, HL7, GraphQL, REST, and semantic approaches to observe more effective and efficient architectural solutions that can be used to achieve interoperability between health information systems for organizational use.
- **Integration with AI, ML, DL, IoT, FL, and edge computing:** BC uses found synergized in healthcare as it enters the fourth stage in development with the intensifying integration of AI [45,98]. Machine learning [115], deep learning [101], federated learning [71,110], IoT [47,48,116], edge computing [52], and cloud computing [51] have all actively been synergized through BC design and development architecture. Researchers are using these technologies to evaluate that BC can enhance the transparency, traceability, and explainability of AI-driven healthcare distributed systems having predictive training capabilities of data intelligence, decision making, and epidemiology [51]. Moreover, leveraging edge computing, a hybrid edge BC-based framework [52] was implemented to facilitate the necessary requirements for a health- care ecosystem in terms of scalability, security, as well as privacy.
- **Decentralized governance:** Furthermore, studies centered on using BC-based implementation in supporting providers of healthcare services with governance tasks, e.g., self-sovereign identity for patient-centric healthcare [117] and fusion identity management into global healthcare [118]. To eliminate a third party and single point of failure, ref. [89] focused on enabling trusted parties only to ensure access to private data. This research centered on how the BC development methods boosted efficiency and added trust, value to established models, and new structures to end governance management.
- **Drug traceability and fraud detection for drug counterfeiting:** The advent of threatening patient safety led to data integrity and financial losses. BC offered transformative solutions to enhance maximum drug transparency and traceability [104], detect fraud effectively [61], enhance prescription fraud avoidance [105], and enhance automatic claim resolution [108], which have all been implemented having a measured provenance for BC in the healthcare supply chain. It further enables all parties to verify the authenticity of a drug’s origin and analyze traceability using BC logs for compliance with regulatory standards; BRUINChain achieved traceability as a 100% success in counterfeit detection; and scalability: 2 k TPS [62]. Moreover, ref. [107] implemented a pharmaceutical supply chain using BC in IoT cloud systems, where IoT devices and smart labels (e.g., QR codes, RFID tags) can be linked to BC systems to log real-time data about the location, temperature, and handling conditions of drugs.
- **Large-scale solutions:** Large volumes of data are being produced by desperate healthcare systems, necessitating secure, scalable, and effective management solutions. ABC implementations [42,108,113] explained promising decentralized medical record self-management systems, with an extent of scalability and transaction throughput attempted with low fees. Additionally, large-scale medical imaging applications PCMIMS [49] and PCRIM [67] validated large patient-centric medical image processing, requesting, and test report management systems through BC schemes. Their results show the decentralized storage and privacy of medical imaging data in a controlled and managed way with patient consent. However, our research witnessed a prominent gap in privacy mismanagement and data tempering while dealing with large-scale implementation. So, we explored Layer-2 solutions to offer a practical means of moving beyond these restrictions, allowing for more practical applications. The benefit of Layer 2: rollups can help update patient data effectively without putting a strain on the main chain so that data records are accessed more quickly while preserving their privacy and integrity.
- **Applications in precision medicine:** Advancing in the use of genetic, environmental safety, and lifestyle health data, precision medicine seeks to customize medical care to each patient’s unique traits. BC provides a strong back for tackling important precision medicine issues such as data security, interoperability, and patient consent. The only two implementations, i.e., 2% from included studies of this SLR, observed the implementation of genomic data management, such as NFT-enabled privacy, ownership genomics protection systems [54], and decentralized genomics audit logging [55]. Their results demonstrate the feasible ledger for genomic query log data yielded with 30–40% increase in insertion efficiency and privacy-preserved genomic data management. However, the secure sharing of genomic data becomes a paramount concern for further research.
- **Regulatory compliance and adherence:** BC’s architecture inherently has a permissions layer to ensure that transactions are secure, authenticated, and verifiable. It further holds the potential to strengthen patient-centricity [85], cross-chain interoperability [48], and data security [93] in the healthcare industry. However, for this implementation to be successful, regulatory compliance frameworks such as the General Data Protection Regulation (GDPR), Electronic Identification, Authentication, and Trust Services (eIDAS), and Health Insurance Portability and Accountability Act (HIPAA) must be complied [114]. Here, the authors implemented a Smart Patient Consent Management model for HIE and achieved excellent traceability, openness, and dependability for sharing patient data in research institutions and hospitals complied with GDPR. Additionally, many BC-based implementations aligned their work with regulations such as GDPR and HIPAA while exploring innovations such as regulatory sandboxes and decentralized governance models. In this context, our research explored the European Blockchain Sandbox [119], which is a promising controlled environment where BC solutions are actively researched, tested, and developed within the framework of EU regulations. It facilitates innovation, collaboration, and compatibility while ensuring compliance with the GDPR, eIDAS, or other applicable laws. This initiative allows stakeholders, including startups, enterprises, and regulators, to collaborate and address challenges in BC adoption.

In conducting this research, few limitations have been observed in the standardization of BC design and development methods due to their exploratory and infancy paradigm. For example, none of the studies implemented any standard framework such as BC layered architecture or Layer 2, sharding, scaling, or tokenization solutions. We also faced some challenges initially in the bias or quality assessment and then in the data extraction synthesis stage; this is due to the large number, i.e., 82 included studies with diversified areas or simply called non-randomized control trials. However, the contribution was promising when we assessed the full text of all studies and explored common correlated fields of relevance to group them as qualitative thematic categorization and quantitative reported wherever applicable.

Moreover in general, we also observed the following limitations:

**Selection bias:** This review focused on five main databases, potentially excluding relevant studies. To address selection bias, future reviews should include an additional 722 databases and sources, such as gray literature (e.g., reports, conference papers), and search a broader range of platforms to include studies not covered by traditional academic databases.

**Exclusion of gray literature**: Excluding gray literature might have overlooked valuable insights from industry reports, news, and technical papers. Future studies or reviews should include such gray sources to capture practical BC applications.

**Practical adoption barriers:** Despite the technological promise of BC in healthcare, we experienced practical adoption barriers such as:

**Regulatory/ethical hurdles:** Different regions have varying regulatory data privacy law (GDPR or HIPPA) frameworks that complicate the widespread adoption of BC-based healthcare solutions.

**Clinician usability and integration:** Healthcare professionals must be able to understand BC technology into their daily practices easily. Usability concerns regarding the complexity of Blockchain interfaces could impede its adoption. Many healthcare institutions rely on third-party-established EHRs, which may not easily integrate with BC technologies. However, their formatting, such as FHIR/hl7, goes as standard. This integration challenge can slow down the transition to decentralized systems.

**Real-world deployment barriers:** Many BC solutions are still in experimental stages, or PoCs or simulated environments, and real-world deployment in clinical settings faces challenges related to scalability, verbality, cost, and security.

**Language bias:** The exclusion of studies not in English can introduce bias. However, we kept a balance in the response process and maintained transparency by developing generic eligibility criteria. However, future work might consider including non-English studies as well to mitigate this bias and gain a more comprehensive understanding of BC applications in healthcare.

## 5. Conclusions

In the rapidly evolving landscape of HIT-leveraged BC interventions, we observed that avenues such as medical records, HIE, and secure patient data sharing gained significant attention from researchers and developers. Our research consists of an increasing number of evident studies on BC-based implementations in eight promising healthcare avenues as summarized in Table 4. Interestingly, in addition to the qualitative recommendations, we also found the quantitative results of the studies, since one of the BC implementations achieved almost a 100% success verification of counterfeit drug detection and 2000 TPS scalability using BC logs to analyze traceability in real-world pharmaceutical data [62]. Therefore, our objective was to systematically analyze the previous literature on the pragmatic shift of BC in healthcare to better understand current challenges, implemented solutions, and future prospects. Our four formulated research questions in the context conclude as RQ1 illustrates year-wise publication flow, distribution of publication venues, and region distribution of author relevance. These visualized the existing outlines of current research on the adoption of BC for healthcare avenues. The RQ2 was established to help researchers better understand the foundation of BC and its work with the prominent healthcare challenges reported by the research over the last five years. We discussed the provenance that BC reserves a fundamental base to solve the challenges raised by healthcare avenues by reporting pragmatic BC-based applications. However, RQ3 follows the list of aspects of strengths and weaknesses analyzed either from robust, validated outcomes of applications or the limits of their methods to contribute to results. This enabled us to understand, identify, and report prominent research gaps in the existing literature. Thus, our last RQ4 explained and summarized the BC design development methods applied by the applications to provide practical solutions to real-world healthcare issues. We also report those applications that utilized real healthcare data or datasets and performed simulation and analysis through evaluation to measure effective outcomes and respective matrices. Overall, BC offers advantages in healthcare, such as improving transparency and patient control, but raises ethical challenges, particularly around patient consent for healthcare or genomic data sharing. BC’s democratic and immutable nature complicates consent management, and ensuring compliance with privacy regulations (e.g., GDPR, HIPAA) is essential. Additionally, while BC promotes transparency, data privacy remains a concern, requiring privacy-preserving technologies such as zero-knowledge proofs to protect sensitive information. This SLR experienced some limitations discussed in later section, but notably selection bias due to the restricted choice of five databases. Excluding other sources such as gray literature (e.g., industry reports and preprints) limits the comprehensiveness of the findings at the implementation level. Our future reviews and studies should include a wider range of sources and databases to address this bias and provide a more complete picture of BC applications in healthcare.

## 6. Challenges and Future Research Direction

Our research rigorously found prospects for BC-based healthcare developments based on extracted data from included studies of this SLR. This research followed a systematic review protocol, which allowed us to explore more peer-published literature, gray literature, published reports, and related news portals. From all sources, we observed a wider range of challenges and pain points facing healthcare stakeholders. The dire need for BC-based personalized medicine has been examined and helps to enable secure sharing of genomic data with researchers and pharmaceutical companies for personalized treatment planning. Their future measurement system might evaluate the speed of data retrieval, compliance with patient consent, and advances in research facilitated by secure data sharing. Secondly, another area for further exploration is the way to deal with global health initiatives through decentralized BCs, which might support universal health systems by offering cross-border data sharing and scalable infrastructures through familiar on-chain methods, such as Layer-2 solutions, sharding, and tokenization. Their evaluation could focus on system scalability, global data integration rates, and equity in health outcomes. Finally, as the hot area for the future, we observed that the synergy between artificial intelligence and BS is at its peak, where BC could validate and secure datasets trained by AI systems for predictive analytics and diagnostics. Its metrics to assess this synergy could include improved model accuracy and data bias reduction rates. In addition to this SLR, the experiment to implement BC in personalized medicine for secure genomic data sharing may be positioned as a future prospect. Based on deep-dive research in the literature, this experiment lays the foundation for future advances in secure genomic data sharing, offering a patient-centric and scalable framework that addresses challenges.

- **Data security and privacy:** Genomic data are highly sensitive, constructed, and valuable; its accessibility and sharing yields risk of breach enabling a prime target for cyber attacks. So, ensuring adherence to regulations such as GDPR, HIPAA, or local laws can be complex, and maintaining data ownership control, anonymity, privacy, and secure genomic data sharing for meaningful access and research poses a significant challenge.
- **Lack of standardization and interoperability:** Diverse data formats, structures, and a lack of standardization in healthcare or genomic data across platforms hinder seamless sharing. Differences in privacy regulations across regions also create barriers to sharing data internationally besides cross-border data exchange. Integrating genomic data with other healthcare datasets (e.g., EHRs) for holistic analysis requires robust standards and protocols.
- **Ethics and consent management:** Patient consent for data usage is often overlooked, and managing clear, comprehensive agreement is essential to obtaining a code of ethics. Data ownership is another persistent challenge that leads to undefined ownership of genomic data—patients, researchers, or institutions. Additionally, potential misuse appears as risks of genetic discrimination or unauthorized use of data for purposes away from initial consent.
- **Scalability and performance:** Genomic datasets are massive structures, and large data volume-based iterations require high storage and computational power. Their real-time access, supporting efficient live queries for research and treatment decisions, is challenging.
- **Trust and transparency:** Patients and even healthcare stakeholders may not fully understand the benefits and risks of secure genomic data sharing. This lack of awareness hinders its adoption and calls transparency into question. Further, there is a trust deficit related to patients’ being reluctant to share their data due to fears of misuse, less transparency, or breaches. Cultural sensitivity is challenging in genomic research and data sharing must respect cultural and societal norms around genetic information.
- **Cost and resources:** Infrastructure setup costs in massive amounts, and maintaining operative secure storage, back-end servers, BC networks, and data processing systems can be expensive too. These high costs may limit access, generate inequity to personalized medicine, and exacerbate health inequities.
- **BC integration technical challenges:** Integrating limitations for BC’s on-chain storage capabilities are insufficient for large-scale genomic datasets, necessitating off-chain decentralized storage such as IPFS. Additionally, the time required for BC transactions might delay critical data retrieval, yielding low latency. Energy consumption is another hurdle for BC systems, especially those using proof of work (PoW), which might be resource intensive.
- **Data integrity and authenticity:** Ensuring verification issues such as shared data remain unaltered and originate from a trusted legitimate source is critical. Implementing auditability mechanisms to track data usage without compromising privacy is complex.
- **Collaboration challenges:** Coordination among stakeholders is unavailable, which means researchers, healthcare providers, and policymakers often operate in silos, which leads to complicating collaborative efforts. Negotiating agreements between institutions for genomic data sharing can delay research and there are no such data sharing agreements due to this challenge.
- **AI and genomics dilemmas:** At any stage, using AI to analyze genomic data can lead to biases in the algorithm, especially if the training datasets are not representative. Understanding AI decision-making processes in genomics interpretation remains a challenge to appraise transparency issues. This is the unique challenge that our research identified, and to address, this we build a novel model for BC-based secure sharing of genomic data using hybrid cryptography, SSI, and encryption techniques. Additionally, our progressive research and development aims to adopt innovative solutions for the pressing need for tailored AI, BC, and large language models (LLMs) solutions that fully address, tame, and advance the specific challenges posed by regional healthcare infrastructures and centers [120,121]. BC applications must be designed to accommodate local variations in data formats, privacy laws, and technological readiness. This adaptability is critical to successfully implement BC in diverse health centers and settings.

## Figures and Tables

**Table 2:** Research questions and rationale.

Research Questions:	Rationale:
**RQ1: What is the current state of BC technology research in healthcare?**	This is a rapidly developing research area, so it would be relevant to obtain an impression of the research published over the past five years, their venues, and origin.
**RQ2: Which fundamental healthcare avenues used BC technology to solve issues raised by earlier studies?**	BC technology revolutionizes new healthcare applications, but studies highlighted many new ventures in the infancy of many healthcare areas. So, it becomes paramount to explain its fundamentals and ways to solve healthcare challenges raised by developments explored by our research.
**RQ3: What are the strengths and weaknesses catered from BC-based healthcare implementations?**	BC-based healthcare implementations offer significant advantages, but they also invoke new methodological limitations. So, an overview of the strengths and weaknesses derived from current research takes a clear picture for real-world uses.
**RQ4: How do BC-based applications,** **their applied research methods, data analysis, and evaluation metrics correlatively contribute to present and possible future prospects for healthcare development?**	Understanding methods, data sources, and respective evaluation emphasizes implementation. So, this part aims to explain BC design development methods, data analysis, and evaluation metrics by reporting those developments that analyzed data as qualitative or quantitative evidence. Here, we also report effective outcomes from the insights of evaluation matrices.

**Table 3:** Eligibility criteria.

Inclusion Criteria (IC):	Exclusion Criteria (EC):
IC1: Original peer-reviewed research articles such as primary research articles and concept articles that specifically address the applications of BC in healthcare core avenues.	EC1: Review articles and secondary analyses of previously published articles that do not bring any new information, book chapters, proceedings from symposiums, commentaries, or articles in magazines.
IC2: English language studies published between July 2019 and July 2024 (last five years).	EC2: Duplicated studies.
IC3: Availability of complete texts in search machines.	EC3: Exclude based on bias/quality assessment criteria.
IC4: Proposed implementations or developments adequately address BC methods and techniques in core healthcare avenues.	EC4: Studies that only propose design, system, model, or framework for BC in healthcare applications without implementation or intervention.

## Supplementary Materials

The following supporting information can be downloaded at: https://www.mdpi.com/article/10.3390/healthcare13091087/s1, Supplementary File S1: Search Queries; Supplementary File S2: Quality Assessment -Scored; Supplementary File S3: A PRISMA_2020_checklist for SLR.
