# Supplementary material for: A Systematic Literature Review for Blockchain-Based Healthcare Implementations
Source: Healthcare (Basel). 2025 May 7;13(9):1087. doi: 10.3390/healthcare13091087 (PMC12071524; doi:10.3390/healthcare13091087)
Supplement: Supplementary file 1 [file healthcare-13-01087-s001.zip › healthcare-3569401-Supplementary/healthcare-3569401-Supplementary-S1 Search Queries.pdf]

ScienceDirect:

blockchain or "distributed ledger" and "health care" or "medical" or "health" or "Interoperability"

IEEEXplore:

blockchain or "distributed ledger" and "health care" or "medical" or "health"

PubMed:

(blockchain OR "distributed ledger") AND (Data Anonymization OR electronic health records OR health information exchange OR medical record OR "healthcare data exchange" OR electronic health records OR "healthcare applications" OR Health Information Interoperability OR FHIR)

Web of Science: ALL=((blockchain OR "distributed ledger") AND ("Data Anonymization" OR "electronic health records" OR "health information exchange" OR "medical record" OR "healthcare data exchange" OR "electronic health records" OR "Health Information Interoperability" "healthcare" OR FHIR))

Scopus:

blockchain or "distributed ledger" and "health care" or "medical" or "health"
