# Supplementary material for: A Systematic Literature Review for Blockchain-Based Healthcare Implementations
Source: Healthcare (Basel). 2025 May 7;13(9):1087. doi: 10.3390/healthcare13091087 (PMC12071524; doi:10.3390/healthcare13091087)
Supplement: Supplementary file 1 [file healthcare-13-01087-s001.zip › healthcare-3569401-Supplementary/healthcare-3569401-Supplementary-S2 Quality Assessment -Scored.pdf]

Study and Year	1. Is the source of the text clearly identified?	2. Does the source have standing in the field of expertise?	3. Are the interests of the relevant population the central focus?	4. Is the stated position the result of an analytical process, and is there logic in the opinion expressed?	5. Is there reference to the extant literature?	6. Is any incongruence with the literature/sources logically defended?	Study Quality
Chelladurai et al., 2021	Yes	Yes	Yes	Yes	Yes	Yes	High
Hylock & Zeng, 2019	Yes	Yes	Unclear	Yes	Yes	No	Medium
Kaur et al., 2022	Yes	Yes	Yes	Yes	Unclear	No	Medium
Jakhar et al., 2024	Yes	Yes	Yes	Unclear	Yes	No	Low
Subramanian et al., 2023	Yes	Yes	Yes	Yes	Yes	Yes	High
Ndzimakhwe et al., 2023	Yes	Yes	Unclear	Yes	Yes	No	Low
Naik et al., 2022	Yes	Yes	Yes	Unclear	Yes	No	Low
Tahmasbzadeh & Kabirirad, 2023	Yes	Yes	Yes	Yes	Unclear	No	Medium
Aljaloud, 2023	Yes	Yes	Unclear	Yes	Yes	No	Low
Singh et al., 2021	Yes	Yes	Yes	Yes	Yes	Yes	High
Zhuang et al., 2020	Yes	Yes	Yes	Yes	Unclear	No	Medium
Gohar et al., 2022	Unclear	Yes	Yes	Yes	Yes	No	Low
Xu et al., 2024	Yes	Yes	Yes	Yes	Unclear	No	Low

Pinto et al., 2022	Yes	Yes	Unclear	Yes	Yes	No	Low
Lee et al., 2020	Yes	Yes	Yes	Yes	Yes	Yes	High
Madine et al., 2021	Yes	Yes	Yes	Yes	Unclear	No	Low
Bhattacharya et al., 2021	Unclear	Yes	Yes	Yes	Yes	No	Low
Rahman et al., 2024	Yes	Yes	Yes	Yes	Yes	Yes	High
Marry et al., 2023	Yes	Yes	Yes	Yes	Unclear	No	Low
Costa et al., 2022	Yes	Yes	Yes	Yes	Yes	Yes	High
Puneeth & Parthasarathy, 2024	Yes	Yes	Yes	Yes	Yes	Yes	High
Yuan et al., 2022	Yes	Yes	Yes	Yes	Yes	Yes	High
Yang & Zhang, 2022	Yes	Yes	Yes	Yes	Yes	Yes	High
Naresh et al., 2021	Yes	Yes	Yes	Yes	Yes	Yes	High
Guduri et al., 2024	Yes	Yes	Yes	Yes	Yes	Yes	High
Madine et al., 2020	Yes	Yes	Yes	Yes	Yes	Yes	High

Carter et al., 2019	Yes	Yes	Yes	Unclear	Yes	Yes	Medium
Sun et al., 2020	Yes	Yes	Yes	Yes	Yes	Yes	High
Ilyas et al., 2024	Yes	Yes	Yes	Yes	Yes	Yes	High
Dakshayini et al., 2024	Yes	Yes	Yes	Yes	Yes	Yes	High
Puranik et al., 2022	Yes	Yes	Yes	Yes	Yes	Yes	High
Jha et al., 2023	Yes	Yes	Yes	Yes	Yes	Yes	High
Pattengale & Hudson, 2020	Yes	Yes	Yes	Yes	Yes	Unclear	Medium
Mohsan et al., 2022	Yes	Yes	Yes	Yes	Yes	Yes	High
Reen et al., 2019	Yes	Yes	Yes	Yes	Yes	Yes	High
Nargis et al., 2023	Yes	Yes	Yes	Yes	Yes	Yes	High
Pulmano & Fernandez, 2024	Yes	Yes	Yes	Yes	Yes	Yes	High

Saidi et al., 2022	Yes	Yes	Yes	Yes	Yes	Yes	High
Haddad et al., 2024	Yes	Yes	Yes	Yes	Yes	Yes	High
Akkaoui et al., 2020	Yes	Yes	Yes	Yes	Yes	Yes	High
Li et al., 2022	Yes	Yes	Yes	Yes	Yes	Yes	High
Zhang et al., 2022	Yes	Yes	Yes	Yes	Yes	Yes	High
Pasha et al., 2024	Yes	Yes	Yes	Yes	Yes	Yes	High
Kastowo et al., 2022	Yes	Yes	Yes	Yes	Yes	Yes	High
Abdeen et al., 2019	Yes	Yes	Yes	Yes	Yes	Yes	High
Zaabar et al., 2021	Yes	Yes	Yes	Yes	Unclear	No	Low
Mani & Prakash, 2022	Yes	Yes	Unclear	Yes	Yes	No	Low
Chenthara et al., 2020	Yes	Yes	Yes	Yes	Yes	Yes	High

Mukherjee et al., 2023	Yes	Yes	Yes	Yes	Yes	Yes	High
Miyachi & Mackey, 2021	Yes	Yes	Yes	Yes	Yes	Yes	High
Nedakovic et al., 2023	Yes	Yes	Yes	Yes	Yes	Yes	High
Wajiha & Patil, 2022	Yes	Yes	Yes	Yes	Unclear	No	Low
Zhan et al., 2022	Yes	Yes	Unclear	Yes	Yes	No	Low
Ma & Zhang, 2024	Yes	Yes	Yes	Yes	Unclear	No	Low
Biswas et al., 2020	Yes	Yes	Yes	Yes	Yes	Yes	High
Shen et al., 2019	Yes	Yes	Yes	Yes	Unclear	No	Low
Kumar & Chand, 2021	Yes	Yes	Yes	Yes	Yes	Yes	High
Lee et al., 2022 (Medical Blockchain)	Yes	Yes	Yes	Yes	Unclear	Yes	Medium
George & Chacko, 2022	Yes	Yes	Yes	Yes	Yes	Yes	High

Wang et al., 2023	Yes	Yes	Yes	Yes	Unclear	No	Medium
Lee & Song, 2021	Yes	Yes	Yes	Yes	Yes	No	Medium
Buzachis et al., 2019	Yes	Yes	Yes	Yes	Yes	No	Medium
Toshniwal et al., 2019	Yes	Yes	Yes	Yes	Yes	No	Medium
Abouali et al., 2022	Yes	Yes	Yes	Yes	Yes	No	Medium
Rai et al., 2022	Yes	Yes	Yes	Yes	Yes	No	Medium
Cernian et al., 2020	Yes	Yes	Yes	Yes	Yes	No	Medium
Jiang et al., 2019	Yes	Yes	Yes	Unclear	Yes	No	Low
Gomasta et al., 2023	Yes	Yes	Yes	Yes	Yes	No	Medium
Wu et al., 2022	Yes	Yes	Unclear	Yes	Yes	No	Low
Hu et al., 2023	Yes	Yes	Yes	Yes	Yes	No	Medium
Puneeth & Parthasarathy, 2023	Yes	Yes	Yes	Yes	Yes	No	Medium
Mangala et al., 2024	Yes	Yes	Yes	Yes	Yes	No	Medium
Rohini et al., 2024	Yes	Yes	Yes	Yes	Yes	Yes	High
Oliveira et al., 2022	Yes	Yes	Yes	Yes	Yes	Yes	High
Smith et al., 2023	Yes	Yes	Unclear	Yes	Yes	No	Low
Lee et al., 2023	Yes	Yes	Yes	Yes	Unclear	No	Low
